# Structural Change in Adult Attachment Insecurity Through Transactional Analysis (TA) Developmental Collage Therapy: A Correlational Analysis

**DOI:** 10.7759/cureus.104177

**Published:** 2026-02-24

**Authors:** Hidemi Nakano

**Affiliations:** 1 Clinical Psychology, Tokyo Clinical Psychology Counseling Academy, Tokyo, JPN

**Keywords:** adult attachment, collage therapy, decontamination, ego states, internal working model (iwm), mentalization, mental operating system (mental os), structural reorganization, transactional analysis (ta)

## Abstract

Background: Internal Working Models (IWMs) are cognitive structures for simulating and predicting relational outcomes. While self-report attachment scales, such as the Experiences in Close Relationships (ECR), effectively measure the emotional and behavioral dimensions of attachment, they do not directly assess the cognitive function that processes attachment-related information. Transactional Analysis (TA) offers the concept of the Adult ego state, defined as the capacity for reality testing and objective information processing. This study examined whether incorporating a measure of Adult ego state functioning alongside attachment assessment could reveal structural relationships not detected by attachment scales alone.

Methods: A total of 31 healthy Japanese adults (7 males, 24 females; mean age = 51.8 years) completed the Experiences in Close Relationships - Generalized Other (ECR-GO) and Tokyo University Egogram (TEG3) before and after participating in a TA-based collage therapy program. Spearman correlations were calculated between ego state scores and attachment dimensions at both time points.

Results: At baseline, a significant negative correlation was observed between Adult (A) and Anxiety, that is, negative self-image (*ρ* = -0.493, *p* = 0.005), indicating that low self-image and low Adult ego state functioning are associated. This correlation disappeared after the intervention (*ρ* = -0.312, n.s.), while a new positive correlation emerged between Adapted Child (AC) and Anxiety, that is, negative self-image (*ρ* = 0.492, *p* = 0.005). Both Anxiety and Avoidance decreased significantly (*p* < 0.0001).

Conclusions: The inverse relationship between Adult ego state and attachment anxiety at baseline, and the subsequent change in correlation patterns, may suggest structural reorganization consistent with the TA concept of decontamination. This study is a correlation-based analysis and does not constitute causal inference. The concurrent use of cognitive function measures and attachment scales can reveal structural relationships that attachment measures alone cannot detect. Further research with larger samples is warranted.

## Introduction

Bowlby [1] defined Internal Working Models (IWMs) as cognitive structures that enable individuals to simulate and predict relational outcomes. By using the term "working model," Bowlby emphasized the cognitive nature of this structure - its function as a mental mechanism for simulation and prediction.

The cognitive nature of IWMs becomes particularly significant when considering adult attachment. According to Piaget [2], humans reach the "formal operational stage" in adolescence and adulthood, acquiring the capacity for abstract thinking, hypothesis formation, and logical reasoning. While infant IWMs function primarily through physiological and emotional regulation, adults who have reached this developmental stage would naturally process attachment-related information through more sophisticated cognitive operations. This suggests that evaluating cognitive function may be relevant to understanding adult attachment.

The Experiences in Close Relationships (ECR) and related self-report scales have become standard instruments in adult attachment research, measuring two dimensions: Anxiety and Avoidance. These scales effectively capture the emotional and behavioral aspects of attachment styles. However, they do not include a direct measure of the cognitive function that processes and regulates these attachment-related responses. This raises the question of whether existing instruments from other fields might complement attachment assessment by providing such cognitive measures.

Transactional analysis (TA), developed by Berne [3], offers a framework for understanding personality structure through the concept of ego states - coherent systems of thought, feeling, and behavior. Berne [3] identified three ego states: Parent (P), Adult (A), and Child (C). The Adult ego state is defined as the capacity for reality testing, objective data processing, and logical reasoning, functionally corresponding to Piaget's formal operational thinking.

Of particular relevance to the present study is Berne’s [3] concept of "contamination," a condition in which the boundary between ego states becomes permeable. Child-to-Adult contamination (C→A contamination) occurs when emotional content from the Child ego state is mistaken for objective reality by the Adult. In this state, subjective feelings such as anxiety are processed as if they were facts, impairing the individual's capacity for accurate reality testing.

Based on the functional definitions outlined above, a correspondence between ego states and attachment dimensions may be proposed: Anxiety (ECR) primarily reflects the Child (C) domain - emotional responses related to fear of abandonment; Avoidance (ECR) primarily reflects the Parent (P) domain - internalized rules about self-reliance. The Adult (A) represents the cognitive function that processes and regulates these responses - a dimension not directly measured by current attachment scales. If Anxiety correlates negatively with Adult scores, this may indicate C→A contamination.

The present study examines the correlations between attachment dimensions (Experiences in Close Relationships - Generalized Other, ECR-GO) and ego state structure (Tokyo University Egogram, TEG3) in a sample of Japanese adults. By presenting correlational data between these two assessment systems, the specific aim of this paper is to explore the potential value of incorporating cognitive function measures alongside attachment style assessment in adult attachment research.

## Materials and methods

Study design and participants

This study is a secondary analysis of data from a therapeutic intervention study originally conducted as doctoral dissertation research at the Graduate School of Art, Kyushu Sangyo University (Shinoki [4]; the author now publishes under the name Nakano). This original research, which also served as the basis for a clinical case report (Nakano [5]), investigated whether collage creation combined with group sharing facilitates life script modification, based on TA theory. During that research, a change in correlation patterns between ego states and attachment dimensions was observed but not fully explained. The present correlation analysis is exploratory in nature; it re-examines these correlational patterns from the perspective of attachment theory. The specific hypotheses were developed after examining the original data, rather than being pre-specified.

Participants were 31 healthy Japanese adults (7 males, 24 females; mean age = 51.8 years, *SD* = 9.8, range: 25-72 years) recruited via convenience sampling from TA training courses. Initially, 33 individuals enrolled in the study, but two withdrew due to difficulty completing the collage tasks, resulting in a final sample of 31 participants. All participants provided written informed consent after receiving a full explanation of the study procedures, including protection of personal information, voluntary participation, and the right to withdraw at any time. The inclusion criteria were adults who provided written informed consent and were not currently receiving psychiatric treatment. The exclusion criteria included the inability to complete the collage process.

Measures

ECR-GO

The ECR-GO [6] is an attachment-style scale that measures self-image and other-image in attachment contexts with generalized others rather than specific attachment figures. It consists of two subscales: Anxiety and Avoidance. The Anxiety subscale measures self-image - the degree to which one views oneself as worthy of love and care; higher scores indicate more negative self-image. The Avoidance subscale measures other-image - the degree to which one views others as trustworthy and responsive; higher scores indicate a more negative other-image. Items are rated on a 7-point Likert scale. ECR-GO is freely available for research purposes.

TEG3

The TEG3 [7] is a standardized self-report instrument developed by the Department of Psychosomatic Medicine at the University of Tokyo. It measures five functional ego states based on TA theory: Critical Parent (CP), Nurturing Parent (NP), Adult (A), Free Child (FC), and Adapted Child (AC). The instrument consists of 53 items with three response options ("Yes," "No," "Neither"). Each subscale yields scores ranging from 0 to 20. The TEG has established reliability and validity in Japanese populations [8] and has been widely used in clinical and research settings. TEG3 is commercially available (Kaneko Shobo, Tokyo, Japan) and was used under a standard research license.

Procedure

Data were collected between September and December 2020. Participants completed the ECR-GO and TEG3 at two time points: before the intervention (baseline) and after the completion of the therapeutic intervention (post-intervention). The intervention consisted of creating six collage works representing different developmental stages over a three-week period, with weekly group sharing sessions conducted via Zoom.

Statistical analysis

Spearman's rank correlation coefficients were calculated to examine the relationships between TEG3 ego state scores and ECR-GO attachment dimensions at baseline. Wilcoxon signed-rank tests were used to compare pre- and post-intervention scores, as the assumption of normal distribution could not be confirmed. Correlation patterns were also examined at post-intervention to assess changes in the structural relationships between variables. All analyses were conducted using IBM SPSS Statistics for Windows, Version 27 (Released 2020; IBM Corp., Armonk, New York), with the significance level set at *p* < 0.05.

## Results

Table 1 presents the descriptive statistics for all measures at baseline and post-intervention.

**Table 1:** Descriptive statistics for TEG3 and ECR-GO (N = 31) TEG3 scores are presented as T-scores (M = 50, SD = 10). ECR-GO scores are raw totals based on a 7-point Likert scale. TEG3 = Tokyo University Egogram Third Edition [7]; CP = Critical Parent; NP = Nurturing Parent; A = Adult; FC = Free Child; AC = Adapted Child; ECR-GO = Experiences in Close Relationships inventory-Generalized Other version [6]; Mdn = Median; 25th, 75th = percentiles. Sources: TEG3 is commercially available (Kaneko Shobo, Tokyo, Japan [7]) and was used under a standard research license. ECR-GO is freely available for research purposes [6].

Measure	Time	M	SD	Min	Max	25th	Mdn	75th
TEG3 (T-scores)								
CP	Pre	52.41	12.92	26.00	76.00	41.20	53.50	64.00
	Post	53.98	14.32	15.00	76.00	43.60	55.80	64.00
NP	Pre	56.55	11.90	34.00	76.00	49.50	56.80	67.00
	Post	57.41	13.10	38.00	76.00	44.50	59.20	69.00
A	Pre	53.84	14.56	30.00	76.00	43.70	51.00	64.70
	Post	55.00	13.35	29.00	76.00	43.70	56.00	64.70
FC	Pre	50.02	11.82	24.00	71.00	41.20	51.10	60.50
	Post	54.07	11.87	15.00	68.00	44.20	56.50	62.20
AC	Pre	53.01	10.86	33.00	76.00	45.00	51.50	59.20
	Post	49.29	10.19	24.00	76.00	42.80	47.20	54.90
ECR-GO								
Anxiety	Pre	51.90	18.86	24.00	95.00	35.00	48.00	68.00
	Post	36.94	14.92	18.00	75.00	26.00	32.00	46.00
Avoidance	Pre	50.65	14.62	27.00	76.00	38.00	49.00	63.00
	Post	45.61	13.02	23.00	73.00	35.00	45.00	53.00

Baseline correlations between ego states and attachment dimensions

Table 2 presents the Spearman correlation coefficients between TEG3 ego state scores and ECR-GO attachment dimensions at baseline. A significant negative correlation was observed between Adult (A) and Anxiety (*ρ* = −0.493, *p* = 0.005). Critical Parent (CP) also showed a significant negative correlation with Anxiety (*ρ* = −0.371, *p* = 0.040). No significant correlations were found between ego states and Avoidance.

**Table 2:** Spearman correlations between TEG3 and ECR-GO at baseline (N = 31) *p < 0.05, **p < 0.01. TEG3 = Tokyo University Egogram Third Edition; CP = Critical Parent; NP = Nurturing Parent; A = Adult; FC = Free Child; AC = Adapted Child.

TEG3	Anxiety		Avoidance	
	ρ	p	ρ	p
CP	-0.371	0.040*	-0.268	0.144
NP	-0.283	0.122	-0.306	0.094
A	-0.493	0.005**	-0.127	0.497
FC	-0.076	0.684	-0.293	0.110
AC	0.221	0.232	-0.062	0.741

Changes in scores from pre- to post-intervention

Table 3 shows the pre- and post-intervention scores for both measures. For ECR-GO, significant decreases were observed in both Anxiety (*p* < 0.0001) and Avoidance (*p* < 0.0001). For TEG3, Free Child (FC) showed a significant increase (*p* = 0.004), and Adapted Child (AC) showed a significant decrease (*p* = 0.024).

**Table 3:** Pre- and post-intervention score changes (Wilcoxon signed-rank test, N = 31) TEG3 scores are presented as T-scores (*M* = 50, *SD* = 10). TEG3 = Tokyo University Egogram Third Edition; CP = Critical Parent; NP = Nurturing Parent; A = Adult; FC = Free Child; AC = Adapted Child; ECR-GO = Experiences in Close Relationships inventory-Generalized Other version.

Measure	Pre-intervention	Post-intervention	p
	M (SD)	M (SD)	
ECR-GO			
Anxiety	51.90 (18.86)	36.94 (14.92)	< 0.0001
Avoidance	50.65 (14.62)	45.61 (13.02)	< 0.0001
TEG3 (T-scores)			
CP	52.41 (12.92)	53.98 (14.32)	0.273
NP	56.55 (11.90)	57.41 (13.10)	0.525
A	53.84 (14.56)	55.00 (13.35)	0.149
FC	50.02 (11.82)	54.07 (11.87)	0.004
AC	53.01 (10.86)	49.29 (10.19)	0.024

Changes in correlation patterns

Table 4 presents the correlations at post-intervention. The significant negative correlation between Adult (A) and Anxiety observed at baseline (*ρ* = −0.493) was no longer significant at post-intervention (*ρ* = −0.312, *p* = 0.087). In contrast, a new significant positive correlation emerged between Adapted Child (AC) and Anxiety (*ρ* = 0.492, *p* = 0.005).

**Table 4:** Spearman correlations between TEG3 and ECR-GO at post-intervention (N = 31) **p < 0.01. TEG3 = Tokyo University Egogram Third Edition; CP = Critical Parent; NP = Nurturing Parent; A = Adult; FC = Free Child; AC = Adapted Child.

TEG3	Anxiety		Avoidance	
	ρ	p	ρ	p
CP	-0.298	0.103	-0.119	0.523
NP	-0.134	0.472	-0.322	0.077
A	-0.312	0.087	0.013	0.946
FC	-0.225	0.224	-0.223	0.228
AC	0.492	0.005**	-0.215	0.244

## Discussion

Interpretation of baseline correlations

The significant negative correlation between Adult (A) and Anxiety, that is, negative self-image, (*ρ* = -0.493) at baseline is the central finding of this study. This correlation indicates that low self-image and low Adult ego state functioning are associated. In terms of TA theory, this pattern is consistent with Child-to-Adult contamination (C→A contamination), wherein emotional content from the Child ego state intrudes into the Adult, impairing objective reality testing.

This finding supports the hypothesized correspondence proposed in the Introduction: that attachment anxiety reflects primarily the Child (C) domain, and that its relationship with Adult (A) functioning can be empirically observed when both dimensions are measured concurrently.

Interpretation of changes in correlation patterns

Ego States as Structural Components of IWM

Bowlby [1] proposed the IWM, borrowing the term from Craik [9], who defined it as representations that enable the evaluation of the probability of certain outcomes as a function of executing certain behaviors. Bowlby [1] emphasized the "working" aspect - these models can be manipulated to find optimal solutions to attachment-related problems.

However, the specific mechanisms of this "working" process remained underspecified. In the present study, I reconceptualize IWM as an interpersonal prediction model - a system that stores accumulated records of past interpersonal interactions and uses them to predict future relational outcomes. This reconceptualization makes explicit what was implicit in Bowlby's original formulation: IWM functions as a prediction engine based on accumulated relational data.

Furthermore, Bowlby did not operationally define the structural components of IWM, leaving a significant gap in attachment theory that has persisted for over 50 years. In a previous study [5], I proposed that Berne's [3] ego states - Parent (P), Adult (A), and Child (C) - can serve as operational definitions of IWM components. Both constructs address internalized representations of past relational experiences, though they emerged from different theoretical traditions. Bowlby's [1] IWM encompasses cognitive-affective models of self and others derived from early attachment experiences. Berne's [3] ego states represent preserved patterns of thinking, feeling, and behaving derived from past experiences.

This functional correspondence allows us to operationalize what has remained abstract. By measuring ego states with TEG3, I can assess the structural components of IWM quantitatively. This operationalization does not claim identity between the constructs but proposes a working framework that enables empirical investigation of IWM structure and change.

Ego States as Memory Systems

A critical question arises: what exactly is stored as IWM components? Berne [3] characterized ego states as "a set of feelings, attitudes, and behavior patterns." Critically, the Parent ego state consists of patterns "that resemble those of a parental figure," while the Child ego state consists of patterns "that are relics of the individual's own childhood." The term "relics" indicates that these patterns are preserved from the past - they are, in essence, memories.

This definition implies that P and C ego states function as memory systems - integrated units that store consistent patterns of thinking, feeling, and behaving from past experiences. In contrast, the Adult ego state is defined as patterns "adapted to the current reality," representing present-moment information processing rather than stored memories.

This distinction has important implications for understanding IWM. The P-C relationship represents the accumulated relational memories that form the basis of interpersonal prediction. A, by processing current reality, accesses and updates these stored P-C patterns to generate predictions about future relational outcomes.

Dual Nature of Ego States

Ego states possess a dual nature that is essential for understanding IWM structure. On one hand, each ego state (P, A, C) functions as an independent element with its own coherent patterns of thinking, feeling, and behaving. On the other hand, ego states simultaneously function as components within a larger system - the personality as a whole.

This dual nature - being both an element and a system component - enables ego states to serve as the building blocks of IWM. As independent elements, P and C each store their respective relational memories. As system components, they interact with each other and with A, forming the dynamic relational patterns that constitute IWM.

The Structure of Relational Memory

The proposal that P and C ego states function as memory systems raises a further question: what is the structure of these memories? Research on implicit relational knowing provides relevant insights.

Lyons-Ruth et al. [10] introduced the concept of "implicit relational knowing" - knowledge about how to conduct relationships that is represented in procedural rather than declarative form. This knowing is encoded in patterns of interaction rather than in symbolic or verbal representations. Importantly, such knowledge operates outside conscious awareness and is not readily accessible to verbal reflection.

Schore [11] emphasized that early attachment experiences are encoded primarily in the right hemisphere, which processes information in a nonverbal, holistic, and imagistic manner. These early relational memories are stored as implicit, procedural patterns rather than as explicit, narrative memories.

These findings align with the TA conceptualization of ego states. The Parent ego state stores patterns derived from parental figures - not as explicit memories of what parents said, but as procedural patterns of how to respond in relationships. The Child ego state stores patterns from one's own childhood - not as narrative autobiographical memories, but as implicit ways of feeling and reacting in relational contexts.

This suggests that IWM, as operationalized through ego states, consists not of declarative content but of relational procedures - automated patterns of interpersonal perception, evaluation, and response. The P-C relationship represents accumulated procedural knowledge about how relationships typically unfold.

Relation to Prior Research: Extending Descriptive Approaches

The conceptualization presented above builds upon, rather than replaces, prior theoretical contributions to attachment research. It is useful to clarify how the present approach relates to existing frameworks.

Prior research on IWM has made important contributions through what may be termed descriptive approaches - characterizing IWM in terms of its functions (e.g., prediction, emotion regulation), content (e.g., representations of self and others), or developmental origins (e.g., early caregiver interactions). These approaches have provided essential conceptual foundations for understanding attachment across the lifespan.

However, descriptive approaches share a common limitation: they characterize IWM through verbal descriptions without specifying measurable structural components. Terms such as "schema," "script," "representation," or "filter" describe what IWM does or contains, but do not identify discrete elements that can be independently measured and whose relationships can be quantitatively assessed.

The present approach extends these contributions by proposing an operational framework. By identifying ego states as the structural components of IWM, this framework enables (1) quantitative measurement of IWM components through standardized instruments (TEG3); (2) detection of structural change through examination of correlational patterns among components; and (3) integration with established theory through the functional correspondence between ego states and attachment dimensions.

This represents a shift from asking "What is IWM?" (descriptive) to asking "What are IWM's measurable components and how do they relate to each other?" (operational). The descriptive contributions of prior research remain valid and valuable; the present approach adds a complementary level of analysis that enables empirical investigation of structural change.

Importantly, this operational framework does not claim to capture all aspects of IWM. The rich phenomenological and clinical insights from descriptive approaches - regarding the subjective experience of attachment, the narrative construction of attachment history, and the therapeutic relationship - remain essential contributions that the present quantitative approach cannot replace. The goal is integration rather than substitution.

Content-Based Versus Structure-Based Change

The distinction between descriptive and operational approaches has important implications for understanding therapeutic change. Most psychotherapy research has focused on what may be termed content-based change - changes in the magnitude of individual variables such as symptom severity, anxiety levels, or self-esteem scores. Pre-post comparisons typically ask, "Did the scores decrease?"

This approach implicitly assumes that therapeutic improvement consists of a quantitative reduction in problematic content while the underlying organizational structure remains constant. The metaphor is adjustment: turning down the volume on a malfunctioning system.

The present study introduces a complementary perspective: structure-based change - changes in the relationships among system components. Rather than asking only whether individual scores changed, this approach asks, "Did the pattern of relationships among components change?" The focus shifts from the elements themselves to the connections between them.

This distinction can be illustrated through an analogy. Consider a mobile hanging above a crib. Content-based change would involve replacing one of the hanging objects with a different one - the structure of connections remains the same, but an element's properties have changed. Structure-based change would involve reorganizing how the objects are connected to each other - even if the individual objects remain identical, the system's organization has fundamentally changed.

In the present study, both types of change were observed. Content-based change: Anxiety and Avoidance scores decreased significantly (Table 2). Structure-based change: The correlation between Adult and Anxiety disappeared, while a new correlation between Adapted Child and Anxiety emerged (Tables 1, 3). These represent qualitatively different phenomena.

The structure-based change observed here - the reorganization of correlational patterns - suggests that the therapeutic process involved more than symptom reduction. The personality system's organizational architecture appears to have been reconfigured. This is consistent with the TA concept of decontamination as a structural change in ego state boundaries, rather than merely a reduction in anxiety intensity.

Phase Transition in Ego State Networks

The pattern of correlation changes observed in this study - the disappearance of one significant correlation and the emergence of another - may be conceptualized as a phase transition in the network of relationships among ego states and attachment dimensions.

In physics and complex systems science, a phase transition refers to a qualitative change in system organization, such as water transitioning from liquid to solid. The system's fundamental structure changes, not merely its quantitative parameters. The shift in correlation patterns - from the association between low self-image and low Adult functioning (A-Anxiety correlation) to the association between low self-image and high Adapted Child functioning (AC-Anxiety correlation) - suggests structural reorganization: negative self-evaluation, which was previously associated with impaired Adult cognitive functioning (contamination), became associated with the Adapted Child domain, while Adult functioning became independent from self-evaluative fluctuations.

This interpretation is consistent with the TA concept of decontamination as a structural change rather than merely symptom reduction. The therapeutic goal in TA is not simply to reduce anxiety but to restore appropriate ego state boundaries - to differentiate the Adult's reality-testing function from the Child's emotional experiences.

Why Both Phenomena Became Observable in This Study

A critical question arises: why were both content-based change (reduced attachment anxiety scores) and structure-based change (reorganized correlations) observable in this study, when most prior research has detected only one or the other?

The answer lies in the dual nature of ego states described in the Dual Nature of Ego States section. Ego states function simultaneously as independent elements and as system components. This dual nature creates the conditions for observing both types of change: (1) As independent elements: Each ego state (P, A, C) can be measured individually through TEG3. Changes in individual ego state scores represent content-based change - modifications in the magnitude of specific psychological functions. (2) As system components: Ego states interact with each other and with attachment dimensions (measured by ECR-GO). Changes in the correlational patterns among these components represent structure-based change - reorganization of the relationships within the personality system.

Most prior research has employed instruments that measure either isolated variables (e.g., symptoms, single constructs) or global outcomes (e.g., overall functioning, quality of life). Such instruments can detect content-based change but cannot reveal structure-based change because they do not measure the relationships among defined system components.

The combination of TEG3 and ECR-GO in the present study created a unique methodological configuration. TEG3 provides measurement of all five functional ego states as a complete system, not isolated variables. ECR-GO provides measurement of the two fundamental attachment dimensions. By examining correlations between these two measurement systems, we could observe not only whether individual scores changed but also whether the pattern of relationships among components changed.

This methodological approach - measuring a complete personality system (ego states) alongside attachment dimensions and examining their correlational structure - appears to be novel in attachment research. The concurrent observation of both content-based and structure-based change was not a fortunate accident but a predictable consequence of the measurement design.

The "Mental Operating System" Hypothesis

The following theoretical framework is presented as hypothesis-generating. The findings of this study, combined with the theoretical framework developed in preceding sections, suggest a hypothesis regarding the functional architecture of IWM. I propose that IWM functions as a "Mental Operating System" (Mental OS) - an interface that mediates between internal psychological processes (ego states) and external relational environments.

The Interface Function

In computer science, an operating system serves as an interface between hardware (internal components) and applications/users (external environment). It translates inputs from the external world into operations that internal components can process and converts internal processing results into outputs that the external world can receive.

Analogously, IWM may function as a psychological interface: (1) Input processing: External relational stimuli (others' behaviors, facial expressions, verbal messages) are translated into internal psychological representations through the P-C memory system; (2) Output generation: Internal states (emotions, needs, intentions) are translated into behavioral responses through the same system; and (3) Prediction generation: Based on accumulated relational memories (P-C), the system generates predictions about others' likely responses.

This interface function corresponds to several established concepts in attachment and developmental theory: (1) Attunement (Stern [12]): The synchronization of internal states between caregiver and infant may be understood as interface calibration - the mutual adjustment of two Mental OS systems; (2) Representation (Bowlby [1]): The stored patterns of how relational inputs are converted to internal states and how internal states are converted to behavioral outputs; and (3) Mentalization (Fonagy et al. [13]): The capacity to perform reverse translation - inferring others' internal states from their external behaviors.

The Adult (A) as System Administrator

Within this framework, the Adult ego state functions analogously to a system administrator or meta-level process that monitors and can modify the interface operations. The A does not directly participate in the P-C relational memory but observes and regulates how the system translates between internal and external domains.

Contamination, in this model, represents a condition where the interface function itself becomes compromised - the A's monitoring capacity is invaded by C content, resulting in misattribution of internal emotional states as external reality. Decontamination restores the A's capacity to accurately distinguish between internal representations and external stimuli.

IWM as Accumulated Relational Experience

If IWM functions as an interface, then its content - what it "knows" about relationships - must be derived from experience. I propose that IWM can be formally expressed as $IWM = \int (relational\ interactions) dt$. That is, IWM represents the integral (accumulation) of relational interactions over developmental time. Each interaction leaves a trace that contributes to the interface's translation patterns. The P ego state accumulates patterns derived from caregivers' behaviors; the C ego state accumulates patterns of one's own responses to those behaviors.

This formulation has important implications: (1) Developmental trajectory: IWM is not fixed at a single point but continuously updated through the integral of ongoing experience; (2) Therapeutic mechanism: Therapy provides new relational experiences that, when integrated, modify the accumulated patterns; and (3) Structural change: The phase transition observed in this study may represent a qualitative reorganization of the accumulated interface patterns - not merely addition of new content but restructuring of how the system processes relational information.

Limitations of This Hypothesis

This "Mental OS" hypothesis remains speculative and requires further theoretical development and empirical testing. The analogy to computer operating systems, while heuristically useful, should not be taken literally - the brain is not a digital computer. Additionally, the mathematical formulation (IWM as integral) is presented as a conceptual framework rather than a quantitatively testable model at this stage. Future research should develop operational definitions and measurement approaches that could test specific predictions derived from this hypothesis.

Implications for mentalizing

The observed pattern of correlations has implications for understanding mentalizing capacity. Fonagy [14] describes mentalizing as the capacity to assume thoughts and feelings in others and in oneself and emphasizes that differentiating internal from external reality is not universal but rather a developmental achievement. He introduces the concept of "psychic equivalence," a mode of functioning in which mental events are experienced as equivalent to events in the physical world in terms of power, causality, and implications. In this mode, internal experiences are equated with external reality.

The baseline negative correlation between A and Anxiety - that is, negative self-image - observed in this study suggests a state resembling psychic equivalence: low self-image and impaired Adult cognitive functioning - responsible for reality-based processing - co-occur. The individual cannot distinguish between subjective emotional experience (anxiety) and objective assessment of external reality. This is conceptually parallel to what Berne [3] termed "contamination," where Child ego state content intrudes into the Adult.

The disappearance of this correlation following intervention suggests that participants developed the capacity to maintain Adult functioning independently of their emotional state. In Fonagy's [14] terms, they achieved the developmental integration necessary for mentalizing: the ability to recognize anxiety as a subjective internal experience rather than as an accurate perception of external threat. The concurrent emergence of a positive correlation between AC and Anxiety - that is, negative self-image - further supports this interpretation: after intervention, low self-image and high Adapted Child functioning are associated, suggesting that negative self-evaluation became appropriately located within the Child ego state, where emotional self-evaluations belong, rather than contaminating the Adult's reality-testing function.

This finding suggests that the TEG3, when used alongside attachment measures, may provide a quantifiable approach to assessing changes in mentalizing-related cognitive structures.

Possible mechanisms and comparison with prior research

Collage Fragments as Transitional Objects

The therapeutic process observed in this study may be understood through Winnicott's [15] concept of the transitional object. Winnicott [15] described the transitional object as occupying an intermediate area between internal psychic reality and external shared reality - a space where the infant is not challenged regarding the object's subjective or objective nature. In TA Developmental Collage Therapy, collage fragments appear to serve an analogous function for adults.

When participants select and place images representing their early developmental experiences, these paper fragments become externalized representations of IWM that were previously implicit and nonverbal. The collage exists simultaneously as an objective artifact (paper and images) and as a carrier of deeply personal meaning. This dual nature allows participants to engage with their attachment history in a manner that is neither purely introspective nor entirely externalized, but occupies the "intermediate area of experience" that Winnicott identified as essential for psychological development.

Prior Research on Memory Reconsolidation

Prior research on IWM has employed descriptive approaches - characterizing IWM in terms of functions (e.g., information processing, emotion regulation) or content (e.g., schemas, scripts, representations). These approaches have provided important conceptual frameworks for understanding attachment, but they remain at the level of verbal description, without specifying measurable structural components. Consequently, while researchers have discussed the IWM "structure" conceptually, no operational definition of structural components has been proposed that would enable quantitative detection of structural change.

Several theoretical frameworks have proposed that therapeutic change involves the modification of emotional memories through reconsolidation processes. Lane et al. [16] proposed an integrated model in which therapeutic change across multiple modalities results from updating prior emotional memories through reconsolidation, incorporating new emotional experiences. Ecker et al. [17] developed "Coherence Therapy," proposing a three-step process of memory reactivation, mismatch experience (prediction error), and new learning that erases the target emotional schema.

These frameworks represent important theoretical contributions to understanding how psychotherapy might produce lasting change. However, they have not reported empirical data demonstrating structural changes in personality systems. Lane et al.'s model [16] was presented as a theoretical integration without original empirical data, while Ecker et al.'s [17] evidence consists primarily of case analyses identifying the proposed sequence in published case reports from various therapy systems.

Dynamic systems approaches to the psychotherapy process have also been proposed. Schiepek et al. [18] applied synergetics concepts to analyze temporal patterns in therapy process data, reporting discontinuous changes (sudden gains) and increased variability preceding therapeutic transitions. These studies have contributed to understanding the nonlinear nature of the psychotherapy process. However, these analyses focused on temporal fluctuation patterns in process variables rather than demonstrating changes in the structural relationships among theoretically defined personality components.

A fundamental methodological consideration concerns the definition of system components. Bowlby [1] defined IWMs as cognitive structures but did not specify their constituent elements. The companion paper [5] proposed that ego states, as defined by Berne [3], may serve as the structural components of IWMs. In contrast, prior research on memory reconsolidation in psychotherapy has not explicitly defined the components of the personality system being modified. Without such a definition, it remains unclear what structural elements are being "reconsolidated" or "reorganized."

Unique Contribution of the Present Study

The present study adopted a different approach from prior research. While previous studies measured either temporal fluctuation patterns (Schiepek et al. [18]) or proposed theoretical models without original empirical data (Lane et al. [16]; Ecker et al. [17]), this study measured the entire personality system using theoretically grounded instruments.

The critical distinction lies in the explicit definition of system components. The present study, building on the theoretical framework proposed in the companion paper (Nakano [5]), operationalized IWM components as ego states measured by TEG3. This allowed examination of changes in the relationships among defined components - that is, structural changes in a system with specified elements. Prior approaches, lacking such a component definition, could not distinguish between changes within an undefined system and changes in the relationships among defined elements.

Specifically, ego states were defined according to Berne's [3] theoretical framework as coherent systems of thought, feeling, and behavior. The TEG3 provides standardized measurement of all five functional ego states, representing the personality system as a whole rather than isolated symptoms or process variables. The ECR-GO measures the two fundamental dimensions of attachment. By examining the correlational structure between these theoretically defined components, this study could detect changes in the relationships among components - that is, structural changes - rather than merely quantitative changes in individual variables.

The disappearance of the A-Anxiety correlation and the emergence of the AC-Anxiety correlation represent changes in the relational structure among personality components. To our knowledge, this is the first empirical demonstration of such structural reorganization in attachment-related therapy research.

Illustration From Collage Productions

The following interpretations are offered as illustrative hypotheses based on a single case, not as generalizable conclusions. The structural changes detected statistically were also reflected in participants' subjective experiences and collage productions. The case reported in the companion paper [5] provides illustrative examples.

**Figure 1:**
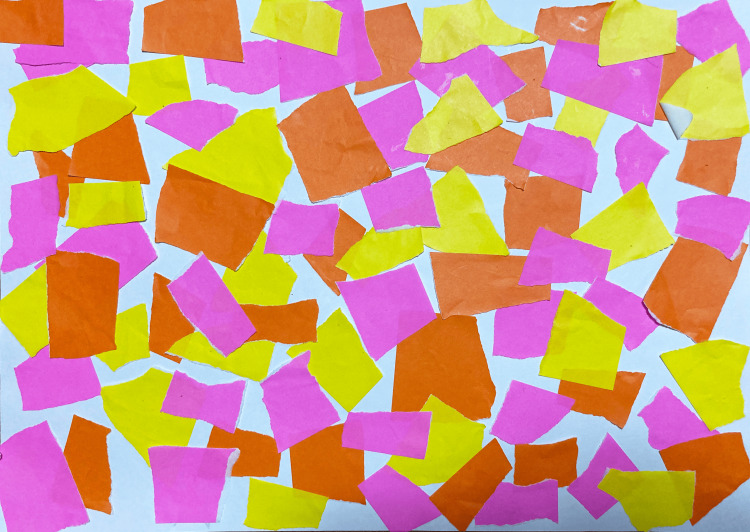
Collage 1 "Warmth" (birth to 18 months) This collage is from the companion case study by the same author (Nakano [5]), published under CC BY 4.0. Yellow, orange, and pink origami paper collaged across the entire surface in a torn-paper style. The participant explained: "Yellow represents mischievousness, orange represents energy, and pink represents love from those around me." In the post-session questionnaire, she reported: "I don't have many memories up to 18 months and don't know about my surrounding environment, but I noticed that the memory of being blessed by everyone remained within me as a bodily sensation and image." This work visually represents the Free Child (FC) ego state and a responsive caregiving environment formed during the preverbal period.

In Collage 1, titled "Warmth" (representing the period from birth to 18 months; Figure 1), the work featured yellow, orange, and pink origami paper collaged across the entire surface in a torn-paper style. The participant explained: "Yellow represents mischievousness, orange represents energy, and pink represents love from those around me." Her verbal report in the post-session questionnaire revealed access to preverbal experience: "I don't have many memories up to 18 months and don't know about my surrounding environment, but I noticed that the memory of being blessed by everyone remained within me as a bodily sensation and image."

The phrase "bodily sensation and image" suggests that attachment-related information stored in implicit, nonverbal form was being accessed and articulated through the collage process. This externalization may represent what memory reconsolidation research identifies as "memory reactivation" - the retrieval of target emotional learning into active awareness. Notably, despite having no conscious memories of this period, the participant could access and express the felt sense of early relational experiences through the nonverbal medium of collage.

**Figure 2:**
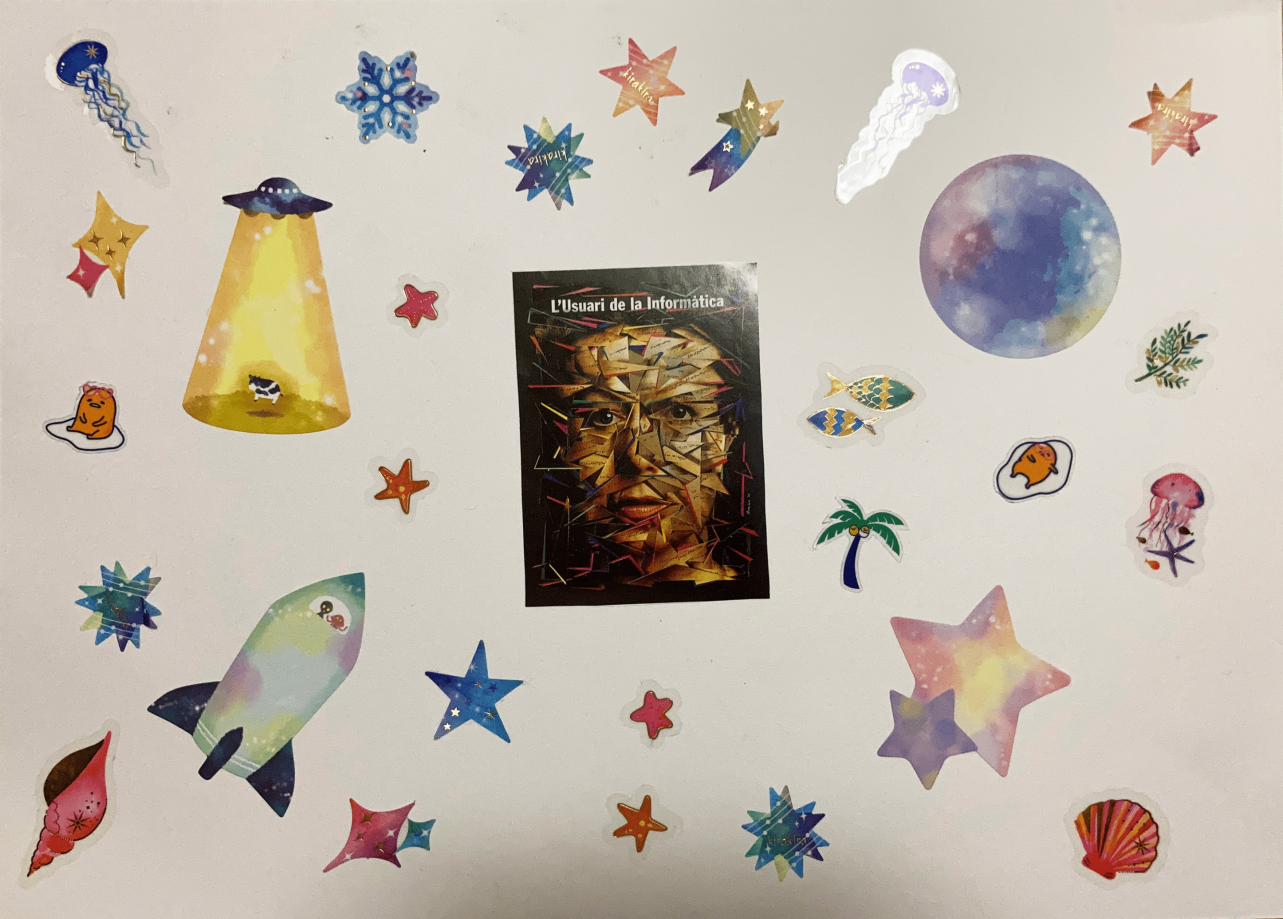
Collage 6 "Freedom" (future afterlife script modification) This collage is from the companion case study by the same author (Nakano [5]), published under CC BY 4.0. At the center, an illustration of a human face is placed, surrounded by space jellyfish, stars, palm trees, rockets, spaceships, and the participant's favorite character. The participant explained: "The face illustration in the center represents myself whose life script is beginning to change, myself being rearranged like a puzzle being reassembled, myself with movement. Around me, fun things are flying around very freely." In contrast to Collages 1-5, where expression remained undifferentiated with torn colored paper, this work features specific objects freely arranged—visually demonstrating the structural reorganization detected in the correlation analyses.

In Collage 6, titled "Freedom" (representing the future afterlife script modification; Figure 2), at the center of the work, an illustration of a human face was placed, surrounded by space jellyfish, stars, palm trees, rockets, spaceships, and the participant's favorite character scattered about. The participant explained: "The face illustration in the center represents myself whose life script is beginning to change, myself being rearranged like a puzzle being reassembled, myself with movement. Around me, fun things are flying around very freely."

This description - "myself being rearranged like a puzzle being reassembled" - corresponds remarkably to the statistical finding of reorganized correlational structure. The participant was not merely reporting symptom reduction but describing an experience of structural transformation in personality organization. The subjective experience of being "rearranged" parallels the objective finding that the pattern of relationships among personality components had changed.

In the post-session questionnaire, the participant further reflected: "This represents not only the surrounding environment but also my inner self. I am expressing that I want to be surrounded by gentle light and animals from inside to outside, and that I want to become peaceful-and I noticed that I want to become that way."

The phrase "not only the surrounding environment but also my inner self" is particularly significant. The collage theme instructed participants to express "myself and my surrounding environment" - precisely the self-other relational structure that constitutes IWM. The participant's recognition that the collage represents both external environment and internal state suggests an intuitive grasp of what the "Mental OS" hypothesis proposes: IWM functions as an interface mediating between internal psychological processes and external relational environments. The shift from the earlier experiences of "darkness" and "walls" (reported in Collages 3-5) to imagery of "gentle light" and "freedom" illustrates the qualitative transformation that occurred through the intervention - a transformation affecting both internal structure and external relational expectations.

Metamorphosis as Metaphor

The transformation observed in this study may be likened to biological metamorphosis, such as a caterpillar becoming a butterfly. In metamorphosis, the organism does not simply grow larger or reduce problematic features; its fundamental architecture is reorganized. Similarly, the change in correlation patterns suggests that the personality system's organizational structure was transformed, not merely adjusted quantitatively.

This metaphor highlights an important distinction: conventional symptom-focused approaches measure whether anxiety decreased (analogous to measuring whether the caterpillar became smaller), while the present approach detects whether the structural organization changed (analogous to detecting whether metamorphosis occurred). Both types of change occurred in this study - anxiety scores decreased (Table 2) and correlation patterns reorganized (Tables 1, 3) - suggesting that structural transformation may underlie symptomatic improvement.

Implications for Developmental Psychology

The findings have implications for understanding adult development. In early childhood, attachment patterns develop through interactions with caregivers who serve as external regulators of the child's emotional states. In adult therapy, the therapeutic relationship and therapeutic tools may serve analogous functions.

The collage process, combined with group sharing, may provide a means through which previously implicit attachment patterns can be perceived and reorganized. The adult's capacity for formal operational thinking - measured here as Adult ego state functioning - may enable more active, conscious participation in this reorganization process than is possible in early childhood. These implications remain speculative and require further investigation.

Need for Further Verification

The interpretations offered above remain speculative and require further verification. The small sample size (*N* = 31) limits statistical power for detecting correlation changes, and the absence of a control group precludes causal inference. The proposed mechanism - that collage work facilitates memory reconsolidation leading to structural reorganization - cannot be directly tested with the present data.

Future research should include larger samples, control conditions, and process measures that can track the proposed mechanisms more directly. Neuroimaging studies examining changes in neural connectivity patterns would provide convergent evidence for the structural reorganization hypothesis. Additionally, replication across different therapeutic modalities would help determine whether the observed pattern of correlation changes is specific to collage therapy or represents a more general signature of structural change in attachment-related therapy.

Significance of incorporating cognitive measures in attachment research

The present findings demonstrate that the concurrent use of TEG3 and ECR-GO can reveal structural relationships that would not be detected by attachment scales alone. Specifically, (1) the association between low self-image and low Adult functioning (A-Anxiety correlation) at baseline provides information about cognitive-emotional structure that ECR-GO alone cannot capture, and (2) the change in correlation patterns from pre- to post-intervention reveals structural reorganization beyond simple score changes.

These observations suggest that incorporating cognitive function measures alongside attachment style assessment may enhance our understanding of adult attachment and its modification.

It should be noted that the Adult Attachment Interview (AAI) assesses "coherence of discourse" as an indicator of attachment security, which indirectly reflects cognitive organization [19]. However, the AAI requires approximately 60-90 minutes for administration [20], and the scoring manual is available only to those who complete official training courses. In contrast, TEG3 is a self-report measure that can be administered alongside ECR in a single session, providing standardized T-scores for cognitive functioning. This practical advantage may facilitate the routine incorporation of cognitive assessment in attachment research and clinical practice.

Limitations

Several limitations should be acknowledged. First, the sample size (N = 31) is relatively small, limiting statistical power and generalizability. Second, participants were healthy adults recruited from TA training courses, not clinical populations, which may limit applicability to clinical settings. Third, TEG3 is a Japanese instrument, and equivalent standardized measures of Adult ego state functioning may not be readily available in other languages. Fourth, the study design does not allow causal inferences about the intervention effects. Finally, the absence of a control group limits conclusions about whether the observed changes are attributable to the intervention itself.

Additionally, participants were recruited via convenience sampling from TA training courses. This recruitment method introduces potential selection bias, as participants likely had higher psychological literacy and prior familiarity with TA concepts than the general population. These factors may limit generalizability to clinical settings and to individuals without such background knowledge.

Future directions

Future research should examine whether similar correlation patterns between cognitive function and attachment dimensions are observed in larger and more diverse samples. The development or adaptation of Adult ego state measures for international use would facilitate cross-cultural research. Additionally, investigating the relationship between Adult ego state functioning and established measures of mentalizing capacity would help clarify the theoretical connections proposed in this study.

## Conclusions

This study examined the correlations between ego state functioning (TEG3) and attachment dimensions (ECR-GO) in 31 healthy adults participating in a TA-based collage therapy program. The central finding was a significant negative correlation between the Adult (A) ego state and Anxiety (i.e., negative self-image) at baseline (*ρ* = -0.493, *p* = 0.005), indicating that low self-image and low Adult ego state functioning are associated. This correlation disappeared after the intervention, while a new positive correlation emerged between Adapted Child (AC) and Anxiety (i.e., negative self-image), indicating that low self-image became associated with high AC functioning. This shift in correlation patterns represents structural reorganization consistent with the TA concept of decontamination.

These findings demonstrate that the concurrent use of cognitive function measures and attachment measures can reveal structural relationships that attachment scales alone cannot detect. Incorporating cognitive function assessment alongside attachment style measurement offers a promising approach for understanding and evaluating changes in adult attachment. Given the limited sample size (N = 31) and resulting constraints on statistical power, these findings should be considered preliminary and require replication with larger samples. Further research with larger samples and direct measures of mentalizing is warranted to confirm these preliminary findings.
